# A case report of pneumopericardium secondary to suspected bronchopericardial fistula post lung resection: diagnosis and management guided by transthoracic echocardiography

**DOI:** 10.3389/fcvm.2025.1673322

**Published:** 2026-01-12

**Authors:** Juan Wang, Zhixin Wang, Meiling Liu, Xijun Zhu, Fei Bian, Qian Liu, Jingjing Cui

**Affiliations:** 1 Department of Cardiology, Binzhou Medical University Hospital, Binzhou, China; 2 Binzhou Medical University, Binzhou, China

**Keywords:** bronchopericardial fistula, cardiac imaging, echocardiography, pericardial disease, pneumopericardium

## Abstract

Pneumopericardium is a rare, life-threatening condition characterized by abnormal gas accumulation in the pericardial cavity, most commonly secondary to trauma, surgical procedures, or fistulous communications with adjacent hollow organs. We report a 59-year-old male patient who presented with chest pain. Initial transthoracic echocardiography (TTE) only detected minimal pericardial effusion, failing to identify pneumopericardium. Subsequent serial TTE monitoring progressively revealed pathognomonic signs of pneumopericardium, including microbubble swirl, air gap artifact, and a definitive fluid-gas level. Notably, the microbubble swirl sign is a typical marker for the early diagnosis of pneumopericardium, and its presence should raise an immediate suspicion of this condition. The diagnosis was ultimately confirmed, and computed tomography (CT) was further performed to corroborate these echocardiographic findings. Therapeutically, ultrasound-guided pericardiocentesis was successfully conducted to drain the pericardial gas. However, post-procedural recurrent pneumopericardium occurred, prompting suspicion of an underlying persistent fistula, specifically a bronchopericardial fistula. Despite aggressive clinical interventions, the patient ultimately succumbed to the disease following voluntary withdrawal of care. This case highlights three key clinical implications: (1) Serial TTE monitoring is of critical value in the dynamic diagnosis of pneumopericardium, particularly when initial imaging yields non-diagnostic results; (2) TTE serves as a dual utility tool—guiding emergent therapeutic interventions (e.g., ultrasound-guided pericardiocentesis) and facilitating etiological investigation (e.g., identifying fistula-related gas recurrence); (3) Clinicians should maintain heightened vigilance for underlying pathological causes (e.g., bronchopericardial fistula) in patients with recurrent pneumopericardium to optimize treatment strategies.

## Introduction

Pneumopericardium refers to the abnormal presence of gas within the pericardial cavity, first documented by Jean-Baptiste Bouillaud in 1844. This condition arises through three principal mechanisms: (1) Direct communication (1): Tracheo-/broncho-pericardial fistulae secondary to malignancy or iatrogenic injury. (2) Pressure gradient-driven: Macklin effect—alveolar rupture with retrograde air dissection along pulmonary vasculature to mediastinum, seen in ventilator-associated barotrauma (incidence: 0.4% in ICU cohorts). (3) Infectious gas production: Clostridial pericarditis with characteristic “crepitus” on autopsy. Notably, tension pneumopericardium, a life-threatening subtype, carries a mortality rate of 50%–57% across etiologies (e.g., iatrogenic pericardiocentesis-related cases, traumatic injuries, or infectious fistula formation) (2, 3). This underscores the critical need for timely diagnosis and intervention.

Pneumopericardium demonstrates five principal etiological categories: traumatic, iatrogenic, adjacent organ pathology, infectious, and spontaneous. Each etiology produces distinct echocardiographic signatures that provide critical diagnostic and therapeutic guidance. Two pathognomonic 2D echocardiographic features are particularly diagnostic: (1) dynamic artifacts characterized by the “swirling microbubble sign”—demonstrating vortex-like movement of hyperechoic microbubbles within the pericardial space synchronized with cardiac cycles, and (2) the “ring-down artifact”—presenting as multiple parallel reverberation lines at the gas-tissue interface. Concurrent anatomical alterations include >5 mm separation of pericardial layers and characteristic sawtooth deformation of the right ventricular free wall, representing mechanical compression by entrapped gas.

For small amounts of air accumulation without clinical symptoms or with mild symptoms, conservative treatment is applicable (4). This includes bed rest, low-flow oxygen inhalation, and avoidance of strenuous activities, and most cases can achieve spontaneous absorption within 1–2 weeks. Massive air accumulation can cause acute cardiac tamponade, leading to a sharp decrease in cardiac output, cardiogenic shock, and even sudden death of the patient (2). In such cases, emergency management is required. Pericardiocentesis for air aspiration is the first choice to quickly expel air, relieve compression on the atria and ventricles, and improve circulatory and respiratory disorders. If necessary, a continuous air drainage channel can be established to prevent recurrent compression. Simultaneously, supportive treatments such as oxygen inhalation and fluid replacement should be administered to maintain electrolyte balance and stabilize vital signs.

Contemporary ultrasound-guided management of pneumopericardium has undergone significant evolution, establishing stratified therapeutic approaches. For hemodynamically stable patients, current protocols advocate high-flow oxygen therapy combined with serial echocardiographic surveillance (recommended every 6–8 h initially). In unstable presentations, emergent ultrasound-guided pericardiocentesis utilizing M-mode trajectory planning and contrast-enhanced microbubble techniques has become the gold standard, significantly reducing complication rates. Video-assisted thoracoscopic pericardial window formation now represents the definitive management for recurrent or complex cases, demonstrating superior outcomes to traditional surgical approaches. The therapeutic armamentarium has further expanded to include innovative techniques such as intrapericardial fibrin glue instillation and closed negative-pressure drainage systems, particularly valuable in high-risk surgical candidates. Importantly, echocardiography serves as the cornerstone throughout the entire clinical continuum—from initial diagnosis (characterizing gas dynamics) to procedural guidance (real-time needle tracking) and post-interventional monitoring (assessing treatment efficacy). This paradigm shift toward image-guided precision medicine continues to redefine minimally invasive strategies in pericardial disease management.

## Case presentation

A 59-year-old male with a history of left lung malignancy resection 14 years prior was admitted for evaluation of worsening chest pain over 15 days, with acute exacerbation in the past 24 h. On examination, the patient appeared acutely ill with a low-grade fever (38.3 °C) and tachycardia (120 bpm). Cardiopulmonary auscultation revealed both a pericardial friction rub and right-sided pleural friction rub. Laboratory tests showed significant inflammatory markers with neutrophils predominating at 85.0% (leukocytosis 14.4 × 10⁹/L, CRP 140.9 mg/L) and elevated BNP (401.7 pg/mL), while ECG demonstrated diffuse ST-segment elevation across anterior and inferior leads (V1–V6, II, III, aVF). The initial working diagnosis was acute chest pain of undetermined etiology, with key differential considerations including infectious pericarditis, post-pericardiotomy syndrome, and myocardial injury.

**Figure 1:**
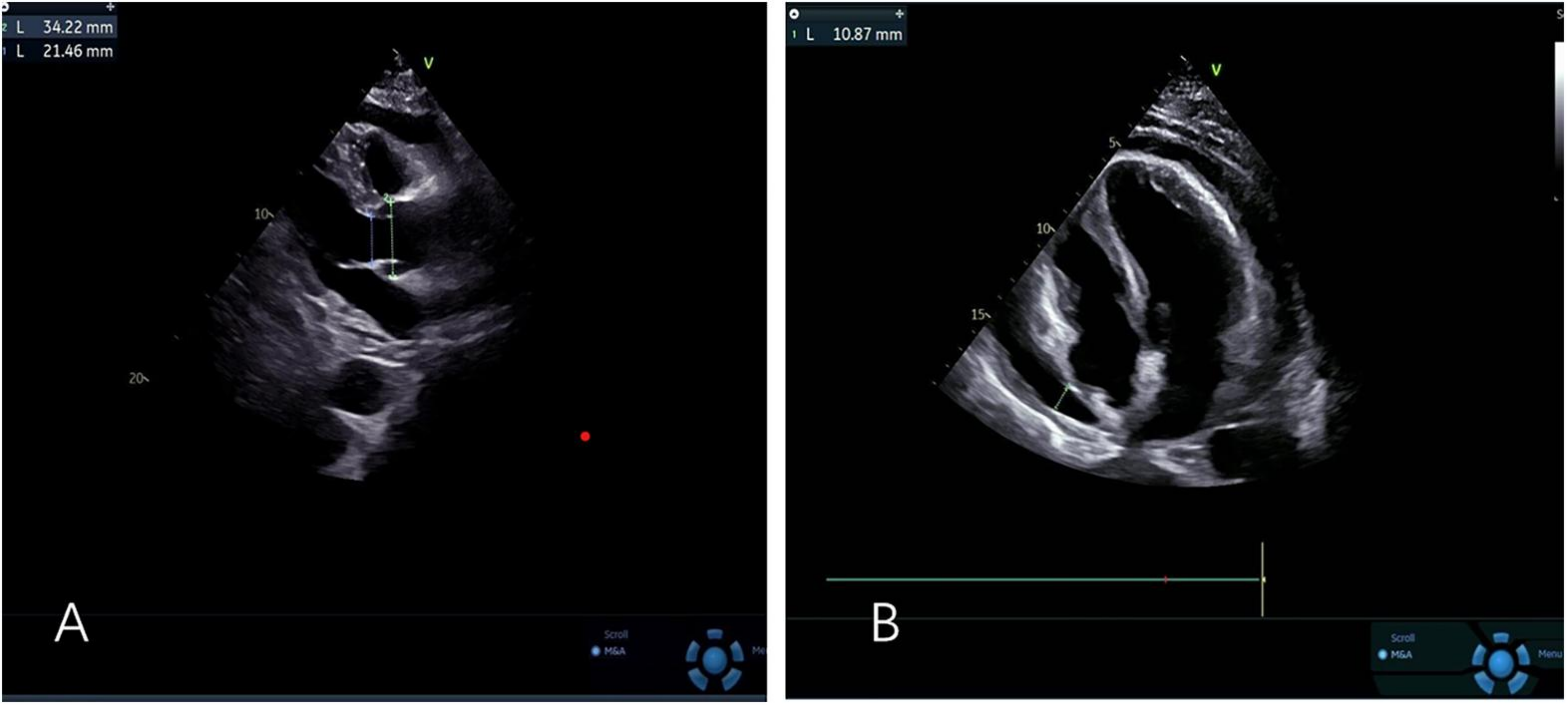
**(A)** Left ventricular long-axis view reveals minimal pericardial effusion **(B)** apical four-chamber view demonstrates mild to moderate pericardial effusion.

**Figure 2:**
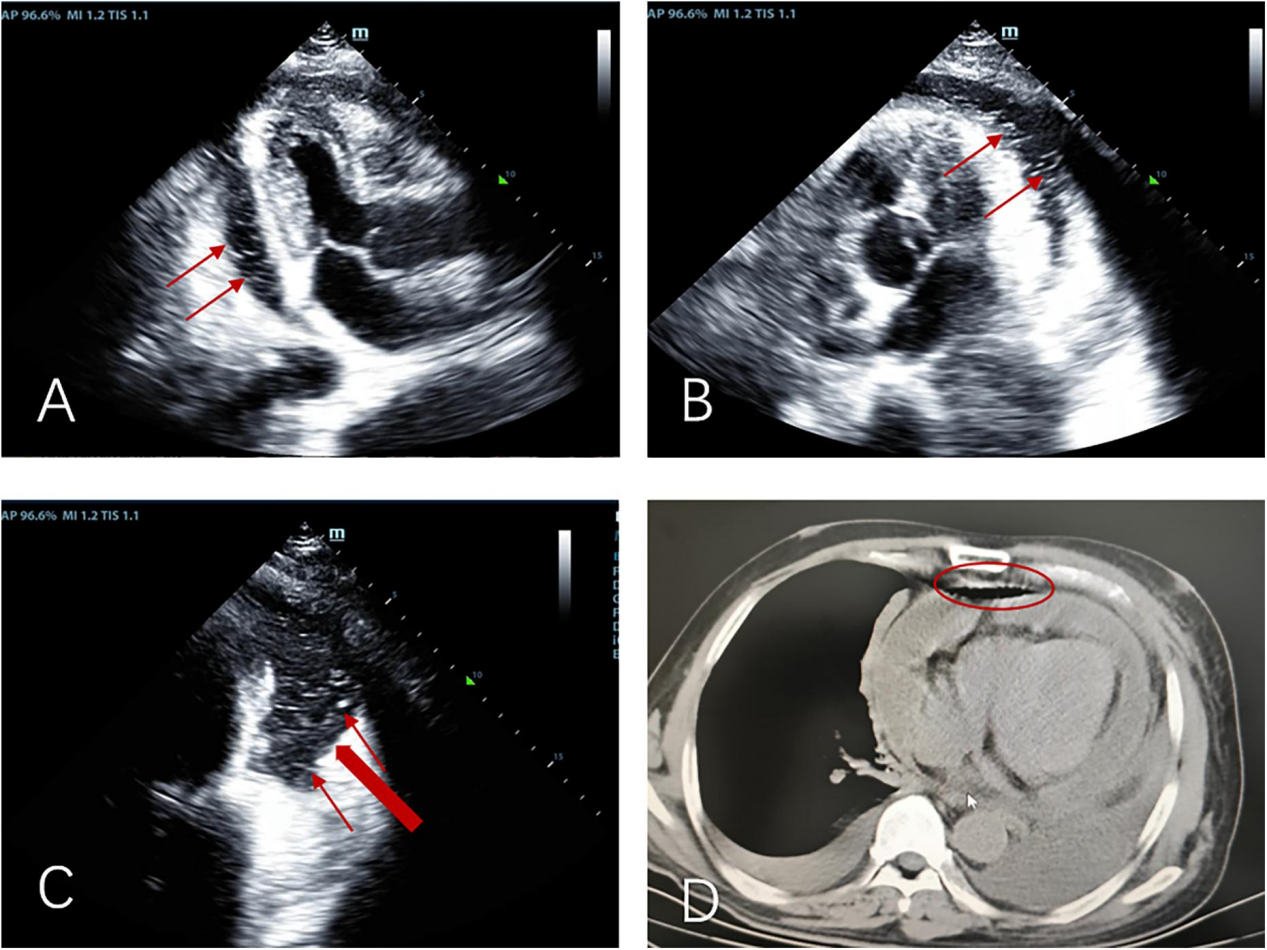
Apical three-chamber view **(A)**, apical five-chamber view **(B)**, and apical four-chamber view **(C)** consistently demonstrate: moderate pericardial effusion with pneumopericardium, “Swirling microbubble sign” (microbubbles originating from the lateral pericardium, thin red arrow), suspected communication between pericardial space and bronchus/residual lung tissue (thick red arrow); **(D)** pre-decompression chest CT demonstrating gas and effusion accumulation in the pericardial cavity.

The patient's initial echocardiogram showed only minimal pericardial effusion (Figure 1A), managed expectantly. Upon admission, ultrasound revealed worsening effusion (14 mm RV anterior wall) with fibrinous strands (Figure 1B). Combined with the patient's signs of low-grade fever, pericardial friction rub and laboratory findings (white blood cell count 14.4 × 10⁹/L, neutrophil proportion 85.0%, CRP 140.9 mg/L), infectious inflammation was highly suspected clinically. Given the rapid progression of the patient's condition, to avoid delaying anti-infective treatment, blood culture or pericardial fluid culture was not performed, and an empirical anti-inflammatory regimen was initiated: anti-inflammatory treatment was administered with Cefoperazone Sodium and Tazobactam Sodium for Injection combined with 40 mg of intravenous methylprednisolone in 100 mL of normal saline. The pivotal diagnostic moment came on day 5 when echocardiography detected rotating microbubbles near the AV groove, suggesting fistulous gas entry—later confirmed by CT as pneumopericardium (Figure 2; Supplementary Movies 1–2).

**Figure 3:**
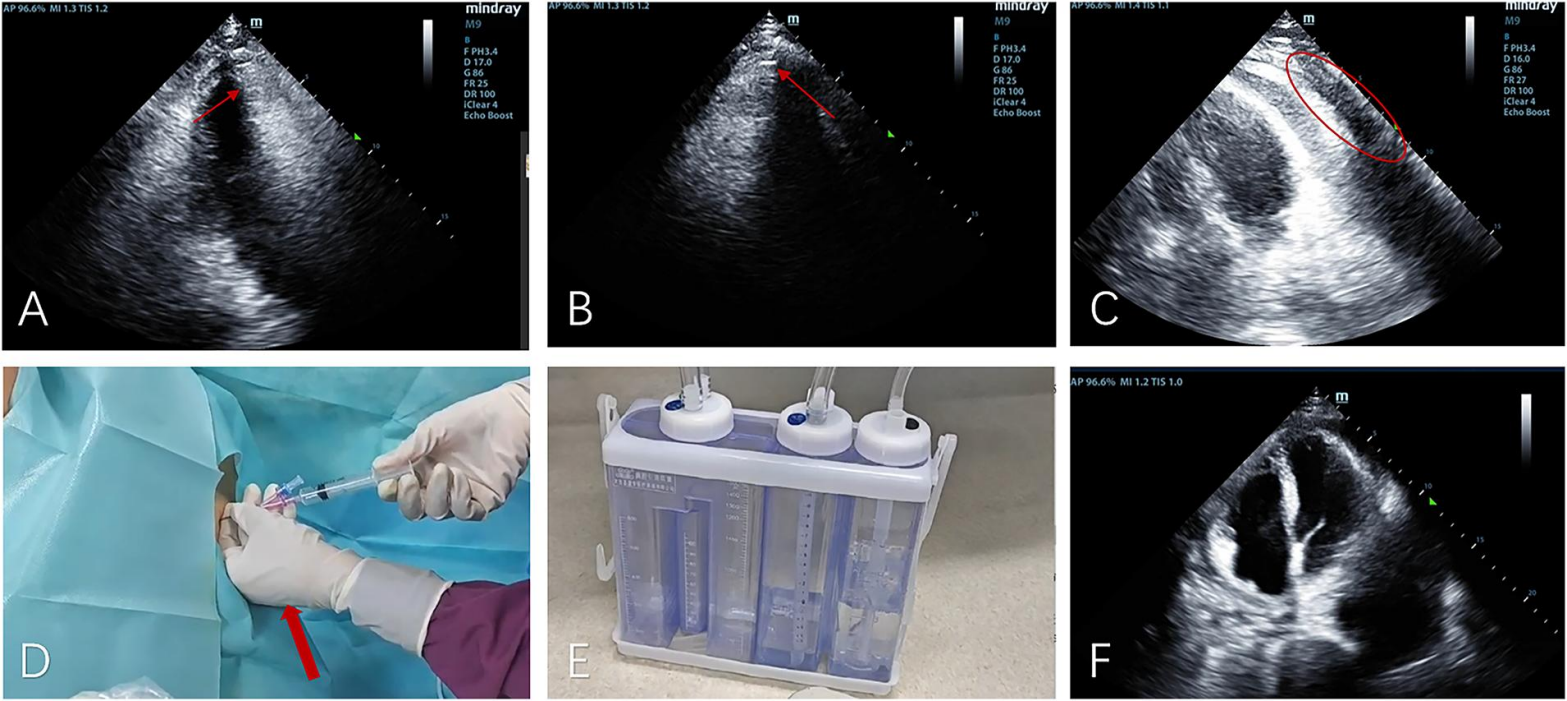
The apical five-chamber view **(A)**, non-standard apical view **(B)**, and apical four-chamber view **(C)** demonstrate superior displacement of the heart with increased intrapericardial gas compared to Figure 2, showing characteristic air-gap artifacts and air-fluid levels (indicated by yellow arrows). Notably, the swirling microbubble density has increased while gas volume decreased (marked by red circles). **(D)** Gas was aspirated from the pericardium (indicated by the red arrow); **(E)** gas was drained into the negative-pressure drainage bottle; **(F)** a reexamination 4 h after drainage showed a significant reduction in intrapericardial gas.

Clinical deterioration occurred abruptly on day 6 following violent coughing: the patient developed acute respiratory distress with pulsus paradoxus. Emergency echocardiography demonstrated dramatic microbubble proliferation, air gap artifacts, and a fluid-gas level (Figures 3A–C; Supplementary Movie 3), confirming tension pneumopericardium. Immediate ultrasound-guided pericardiocentesis (Figure 3D) was performed in the sitting position (2nd intercostal space): approximately 100 mL of gas was successfully drained during this procedure, after which a closed thoracic drainage system was placed for continuous pericardial drainage lasting 6 h (Figure 3E; Supplementary Movie 4).

Post-procedural echocardiography showed significant resolution of intrapericardial gas (Figure 3F), confirming effective decompression.

However, gas reaccumulation within 6 h of drain clamping confirmed persistent fistulation. The temporal association with coughing, anatomical localization of bubbles, and surgical history collectively indicated bronchopericardial fistula. Due to the patient's critical condition and severe dyspnea, it was not feasible to conduct enhanced CT scans or other examinations outside. Subsequently, the patient's blood oxygen levels could not be maintained, necessitating tracheal intubation and tracheoscopy. The tracheoscopy revealed a large amount of yellow purulent sputum in the airway, with yellow purulent secretions and bubbles emerging from the residual left pulmonary bronchus. After receiving anti-infection and nutritional support, the patient still had persistent fever and decreased blood pressure, and the vital signs could not be maintained. The patient was accompanied by mottled skin on limbs. The possibility of refractory septic shock was considered. At this time, the patient's circulatory and respiratory functions were on the verge of failure, and there was no opportunity for surgical repair of the bronchopericardial fistula. After explaining the condition to the family, the patient gave up treatment. After the family opted for withdrawal of medical care, the patient died within two weeks. This case exemplifies echocardiography's critical role in diagnosing dynamic gas accumulation patterns and guiding life-saving interventions in this rare but lethal condition.

## Discussion

This case clearly demonstrates the echocardiographic evolution of pneumopericardium, offering crucial diagnostic insights (Table 1): the early stage presents with the “microbubble swirl sign”—where cardiac motion drives turbulent microbubble movement within the pericardial space, indicating initial gas entry; the progressive phase shows the “air gap artifact” (a circumferential hyperechoic band during systole that obscures cardiac structures); the critical stage develops a definitive “fluid-gas level” in the upright position, signaling impending tamponade from tension pneumopericardium. This dynamic imaging sequence emphasizes that detecting “swirling microbubbles” in pericardial effusion should immediately prompt suspicion of gas accumulation, requiring comprehensive multi-view echocardiographic assessment to localize the gas source and guide urgent intervention.

**Table 1:** Summary of echocardiographic markers for pericardial effusion at different disease stages.

Stage	Marker	specific manifestations	clinical significance
Early stage	Microbubble Swirl Sign	“Microbubble Swirl Sign” (5): Cardiac motion causes turbulent movement of microbubbles within the pericardial cavity	Indicates initial gas accumulation in the pericardial cavity, serving as a key basis for early diagnosis of the disease
Progressive phase	Air Gap Artifact	“Air Gap Artifact” (6): A circumferential hyperechoic band appears around the heart during systole, obscuring part of the cardiac structures	The gas is small and does not produce substantial resistance to cardiac filling (7). Signifies increased gas volume in the pericardial cavity and disease progression, requiring close monitoring of the patient's condition
Critical stage	Fluid-Gas Level	A clear fluid-gas interface is visible in the pericardial cavity when the patient is in an upright position	Indicates the formation of tension pneumopericardium and impending cardiac tamponade, necessitating urgent clinical intervention

Pneumopericardium arises from diverse etiologies that can be classified into five major categories (8): traumatic causes (including penetrating or blunt thoracic injuries) (9), iatrogenic factors (such as post-cardiac surgery or interventional procedures) (10, 11), adjacent organ pathology (e.g., malignant infiltration or fistulous tract formation) (12), infectious origins (notably rare gas-forming bacterial infections), and spontaneous occurrences (typically due to barotrauma from severe coughing or similar mechanisms) (13). Among the aforementioned etiologies, fistula formation caused by adjacent organ pathology is relatively rare, and the occurrence of bronchopericardial fistula 14 years after pulmonary malignancy resection in particular exhibits significant clinical particularity.

In this case, echocardiography played a decisive role in the etiological diagnosis of pneumopericardium and its underlying cause, by identifying four core, mutually corroborative findings that collectively establish the causal link between recurrent pneumopericardium and bronchopericardial fistula. First, serial echocardiographic monitoring tracked the origin of microbubbles to the lateral atrioventricular groove—a region anatomically contiguous with the residual left bronchial stump, a spatial correspondence that aligns with the anatomical basis for fistulous communication between the bronchial tree and pericardial cavity. Second, pericardial gas underwent rapid reaccumulation within 6 h of pericardiocentesis and drain clamping, a kinetics pattern incompatible with physiological spontaneous gas absorption and thus indicative of a persistent pathological gas influx pathway. Additionally, exacerbation of critical symptoms (acute respiratory distress accompanied by pulsus paradoxus) demonstrated a strict temporal correlation with episodes of violent coughing, a clinical association wherein elevated intrathoracic pressure during coughing drives gas shunting through the fistula into the pericardial space. Simultaneously, bronchoscopic examination revealed purulent secretions and air bubbles overflowing from the residual left bronchus, providing invasive endoscopic evidence that indirectly corroborates the presence of an abnormal communication between the airway and pericardial cavity.

Combined with the patient's 14-year history of left lung malignancy resection, these multi-modal findings—encompassing dynamic echocardiographic features, invasive endoscopic evidence, and clinical symptom correlation—collectively and strongly supported a diagnosis of bronchopericardial fistula, rendering this case a paradigmatic example of cross-modal imaging-clinical integration in the management of rare, life-threatening pericardial emergencies. These observations further highlight that for post-pulmonary resection patients presenting with chest pain and coughing-triggered acute dyspnea, the detection of pericardial effusion with dynamically evolving, anatomically localized microbubbles and recurrent pneumopericardium following pericardiocentesis on echocardiography should prompt bronchopericardial fistula to be prioritized in the differential diagnosis, thereby avoiding delays in intervening during the critical therapeutic window for tension pneumopericardium.

Echocardiography demonstrates distinct advantages not only in diagnostic evaluation but also in guiding therapeutic decision-making. For pericardiocentesis localization, it enables real-time identification of the most gas-dense regions (in this case, adjacent to the left atrial roof), ensuring procedural precision (14). In treatment efficacy assessment, post-drainage echocardiographic changes (such as resolution of air-fluid levels) provide direct visual confirmation of therapeutic success (15). Regarding risk stratification, the emergence of sonographic signs including the “gas gap sign” and air-fluid levels—coupled with hemodynamic alterations—serves as an early warning system for impending cardiac tamponade, thereby facilitating timely clinical intervention. This integrated value was fully validated in the clinical management of the present case: such risk stratification guided the urgent ultrasound-guided pericardiocentesis at the critical stage of tension pneumopericardium, and post-drainage resolution of air-fluid levels directly verified the therapeutic efficacy, fully demonstrating the core role of echocardiography throughout the entire diagnosis-treatment process from diagnostic stratification to therapeutic evaluation.

After clarifying the diagnostic and therapeutic value of echocardiography, the mechanism underlying the delayed formation of bronchopericardial fistula in this case requires further analysis combined with the patient's medical history and relevant literature. Based on clinical context and published evidence, three potential pathogenic pathways for delayed fistula formation are proposed as follows. Long-term low-grade chronic inflammation of postoperative pulmonary scar tissue can gradually erode the bronchopericardial fibrous barrier, eventually leading to fistula formation (16). Another plausible pathway involves microscopic residual tumor lesions at the bronchial stump, which may experience late recurrence or invasive progression to penetrate the pericardial tissue and form abnormal communications (17). Additionally, if the patient had received thoracic radiotherapy postoperatively, radiation-induced fibrosis and tissue necrosis can persist for more than a decade, with progressive damage to adjacent bronchial and pericardial structures also contributing to fistula development (18). It should be noted that the aforementioned mechanisms are all speculations based on published literature, and the present case lacks direct pathological evidence due to objective limitations. Due to the patient's critical condition before death, we failed to complete enhanced CT, PET-CT and other imaging examinations to confirm tumor recurrence; meanwhile, because the family refused autopsy, tissue pathology to confirm radiation necrosis could not be obtained. However, the persistent low-grade fever, significantly elevated inflammatory indicators and purulent secretions under bronchoscopy during the patient's course of disease suggested local chronic infection complicated with tissue necrosis, providing indirect evidence for delayed fistula formation.

This study has several limitations to acknowledge. First, as a single case report, its findings lack statistical generalizability; the patient's clinical manifestations, treatment responses, and outcomes may not represent those of the broader pneumopericardium population, particularly individuals with different etiologies. Second, pathological evidence confirming the suspected bronchopericardial fistula is lacking—while echocardiographic findings and clinical context strongly suggested this diagnosis, definitive verification via surgical exploration or histopathological examination was not feasible due to the patient's critical condition and heightened perioperative risk. Third, the patient's follow-up period was limited, precluding assessment of long-term outcomes such as pneumopericardium recurrence, late complications of closed negative-pressure drainage, or the long-term patency of the suspected fistula. Finally, the therapeutic strategy of ultrasound-guided pericardiocentesis combined with closed negative-pressure drainage was tailored to this specific patient and lacked a control group.

To guide the clinical management of pneumopericardium—especially for high-risk populations and in settings with limited evidence—integrated clinical recommendations and echocardiographic diagnostic insights, drawn from expert experience and case observations, are outlined below: When suspecting pneumopericardium on echocardiography, key findings include “air artifacts” (linear or punctate hyperechoic foci) in the pericardial cavity, often accompanied by posterior acoustic shadowing or reverberation artifacts; critically, these foci move synchronously with the cardiac cycle—a feature that distinguishes them from pleural air (which shifts with respiration). Clinically, a high index of suspicion is warranted for patients with risk factors (e.g., lung resection, thoracic trauma, gas-forming infections) or warning symptoms (new-onset chest pain, dyspnea, paroxysmal severe cough, hypotension), as the latter may indicate early hemodynamic compromise.

Emergency pericardiocentesis is indicated if pneumopericardium is confirmed alongside evidence of cardiac tamponade, specifically: tamponade-induced hemodynamic instability (e.g., persistent hypotension unresponsive to fluid resuscitation), large-volume pneumopericardium with ultrasound-confirmed cardiac compression (e.g., right atrial/ventricular diastolic collapse), or persistent or progressive symptoms despite conservative management (e.g., oxygen therapy, bed rest).

Serial monitoring and follow-up should be individualized based on patient status: For stable patients (no signs of tamponade, normal vital signs), initial echocardiographic monitoring is recommended every 4–6 h using a multi-view approach (parasternal long-axis, apical four-chamber, subcostal views); the interval may be extended to 8–12 h if gas volume remains stable or decreases. For unstable patients (e.g., fluctuating vital signs, worsening symptoms), continuous bedside echocardiography is necessary to detect early signs of tamponade. For high-risk populations (e.g., post-lung resection patients), baseline transthoracic echocardiography (TTE) should be performed 1–3 months postoperatively, followed by annual TTE for 3–5 years; immediate additional TTE is required if warning symptoms or events that increase intrathoracic pressure occur. For patients who have undergone pericardial decompression, TTE reexamination is recommended 24–48 h postoperatively, with weekly follow-up TTE until pneumopericardium resolves, and a final reevaluation 1–3 months postoperatively to confirm complete recovery.

## Conclusions

This case confirms that echocardiography can achieve early diagnosis of pneumopericardium, etiological identification, and image-guided pericardiocentesis through dynamic monitoring of characteristic imaging signs; meanwhile, the multi-modal assessment model combining echocardiography and bronchoscopy has provided crucial support for the diagnosis of rare bronchopericardial fistula and established a new paradigm for the diagnosis and treatment of pericardial emergencies in high-risk populations.

Clinical Recommendation: For high-risk populations (e.g., post-lung cancer resection), routine echocardiographic screening for “pericardial microbubbles” should be implemented to detect fistulous tract formation at an early stage, thereby improving patient outcomes.

Core Diagnostic Sign: The microbubble swirl sign is a specific marker for the early diagnosis of pneumopericardium, appearing earlier than air gap artifacts. It is characterized by vortex-like movement of hyperechoic microbubbles in the pericardial cavity synchronized with the cardiac cycle, and its origin site can assist in localizing the fistula (e.g., lateral atrioventricular groove origin suggests potential bronchopericardial fistula).

Clinical Management Principle: Once this sign is detected, immediate serial TTE dynamic monitoring should be initiated to alert for progression to tension pneumopericardium. Simultaneously, infection markers, endoscopic or imaging evaluations should be completed to identify the underlying fistula etiology.

## Data Availability

The raw data supporting the conclusions of this article will be made available by the authors, without undue reservation.

## Supplementary material

Supplementary Movie 1 Pericardial effusion and microbubble sign were observed in the four-chamber view of the apex. Supplementary Movie 2 The intrapericardial microbubbles originate from the pericardium outside the left atrioventricular groove. Supplementary Movie 3 ring-shaped gas sign and fluid-air level in pericardium Supplementary Movie 4 After 5 h of puncture drainage, the gas disappeared.
